# Number of Primordial Follicles in Juvenile Ringed Seals (*Pusa hispida*) from the Gulf of Bothnia and West Greenland

**DOI:** 10.3390/ani12050669

**Published:** 2022-03-07

**Authors:** Britta Schmidt, Julia Hollenbach, Christian Mühlfeld, Christiane Pfarrer, Sara Persson, Tina Kesselring, Christian Sonne, Frank Rigét, Rune Dietz, Ursula Siebert

**Affiliations:** 1 Institute for Terrestrial and Aquatic Wildlife Research, University of Veterinary Medicine Hannover, Foundation, Werftstr. 6, D-25761 Büsum, Germany; tinakesselring@aol.de (T.K.); ursula.siebert@tiho-hannover.de (U.S.); 2 Institute of Anatomy, University of Veterinary Medicine Hannover, Foundation, Bischofsholer Damm 15, D-30173 Hannover, Germany; julia.hollenbach@tiho-hannover.de (J.H.); christiane.pfarrer@tiho-hannover.de (C.P.); 3 Institute of Functional and Applied Anatomy, Hannover Medical School, Carl-Neuberg-Str. 1, D-30625 Hannover, Germany; muehlfeld.christian@mh-hannover.de; 4 Department of Environmental Research and Monitoring, Swedish Museum of Natural History, P.O. Box 50007, SE-104 05 Stockholm, Sweden; sara.persson@nrm.se; 5 Unit for Reproductive Medicine, University of Veterinary Medicine Hannover, Foundation, Bünteweg 2, D-30559 Hannover, Germany; 6 Department of Bioscience—Marine Mammal Research, University of Aarhus, Frederiksborgvej 399, P.O. Box 358, DK-4000 Roskilde, Denmark; cs@ecos.au.dk (C.S.); rdi@ecos.au.dk (R.D.); 7 Department of Ecoscience, University of Aarhus, Frederiksborgvej 399, P.O. Box 358, DK-4000 Roskilde, Denmark; ffr@ecos.au.dk

**Keywords:** ringed seal, primordial follicles, stereology, Baltic Sea, Greenland

## Abstract

**Simple Summary:**

Primordial follicles are the first out of three stadiums of developing follicles in the female reproductive cycle. It is thought that primordial follicles represent the total number of oocytes. That is why we investigated 52 ovaries from ringed seals (*Pusa hispida*) to provide reference data of the total primordial follicle number of juvenile ringed seals. We compared 46 ovaries from the Gulf of Bothnia and six from West Greenland. All ovaries were cut into 2 mm thick slices, embedded in paraffin and stained. We calculated the estimated primordial follicle number by using stereology to estimate the different volumes of the ovary. The median of primordial follicles seemed to be higher in the Gulf of Bothnia ringed seals than in Greenland individuals. Our findings make it difficult to calculate one total range of primordial follicles in ringed seals, but it could be of importance for the identification of differences between populations and for the identification of possible influencing environmental factors.

**Abstract:**

Primordial follicles are important for the reproduction cycle and, therefore, also for the survival of the whole population of a species. Mammals have a large pool of primordial follicles, and it is thought that this pool represents the total number of oocytes. The aim of the present study was to determine the total primordial follicle number of juvenile ringed seals (*Pusa hispida*) from the Gulf of Bothnia and Greenland. Overall, 52 ovaries from two ringed seal populations (West Greenland (N = 6), Gulf of Bothnia, region in the Baltic Sea (N = 46)) were examined. All ovaries were cut into 2 mm thick slices and every slice was embedded in paraffin. Out of each tissue block, a 5 µm thick section was cut and stained with haematoxylin-eosin. The mean volume of the follicles and the total volume of primordial follicles per ovary were estimated by stereology and used to calculate the total estimated number of primordial follicles. The median of the total estimated number of primordial follicles seemed to be higher in Baltic individuals than in Greenland individuals (Gulf of Bothnia = 565,657; Greenland Sea = 122,475). This widens the total range of primordial follicles in ringed seals overall and might bear some potential for discussions regarding the influence of endocrine disruptors and environmental influences depending on different regions/populations and their exposure to various factors. Thus, this study aims to provide basic reference data of the number and mean volume of ringed seal primordial follicles.

## 1. Introduction

Mammalian ovaries have a large pool of primordial follicles [1,2]. Different species develop this pool at different stages of development. In primates and ruminants, the development happens during the early neonatal period, whereas in rodents and rabbits, it develops during the early postnatal period [2,3,4,5]. A primordial follicle consists of an oocyte arrested in prophase 1 of meiosis [1]. Each primordial follicle will go through folliculogenesis, where it first develops into a primary and secondary follicle with support from granulosa and precursor theca cells [2,6]. Granulosa cells form the microenvironment for the oocytes and together with the theca cells, they synthesize a number of hormones, which control follicular development [2]. The tertiary, or antral, follicle contains follicular fluid where the oocyte undergoes maturation. Only tertiary follicles can respond to specific signals and ovulate, for the ringed seals (*Pusa hispida*) one in every normal oestrus cycle [7]. Consequently, only a few primordial follicles successfully reach ovulation and the majority undergo atresia [1]. In this way, the pool of primordial follicles decreases throughout life and menopause occurs when the pool is exhausted [8]. In the Baltic ringed seal, ovulation rate and pregnancy rates (measured as postpartum corpus albicans) decrease in old females, somewhen after the age of 20 years [9].

Disrupted formation of the primordial follicle pool during development or increased atresia at different follicular stages can induce a premature ovarian insufficiency and affect fertility [10]. Known causes of depleted ovarian reserve are e.g., genetic, autoimmune, or related to maternal nutrition, and increasing evidence points to exposure to anthropogenic chemicals such as PCB, PFOS, and phthalates [10,11,12], which can be found in, for example, human follicular fluid [13]. Some physiological parameters are determined by adaptions of the species to certain factors, for example the increased number of primordial follicles in larger species, which can be an adaption to a longer “adult life span” [14].

In this study, the focus is on ringed seals, for which nothing is known about the pool of primordial follicles. Additionally, for all pinnipeds the exact number of primordial follicles is not known. The ringed seal is distributed all over the northern hemisphere [15]. In the Baltic Sea, a subspecies named *Pusa hispida botnica* occurs [16]. This subspecies is found in the Baltic Sea in the following areas: Gulf of Bothnia, Finland, and Riga [17,18]. In the 1970s, the Baltic ringed seal population decreased as a result of anthropogenic pollutants, mostly polychlorinated biphenyls (PCB) and dichlorodiphenyltrichloroethane (DDT) [19]. PCB and DDT have negative effects on the health status of the ringed seals [20], resulting in endocrine disruption, uterine occlusions and stenoses, bone lesions, reduced immune competence or incidence of neoplasia [21,22,23,24,25]. All these conditions affect the overall health status of the seals, which can influence reproductive success, low blubber reserves, and stress [12,18,26].

The aim of this study was to determine the total number of primordial follicles in ringed seals, and for pinnipeds for the first time, which are given at birth to provide reference data for future investigations on the effects of different environmental factors, such as pollution. For this purpose, two ringed seal populations (Gulf of Bothnia and Greenland) were examined with the aim 1—of establishing a suitable morphometric protocol to analyze the delicate ovaries of marine mammals, 2—of providing reference data of the number and mean volume of ringed seal primordial follicles.

## 2. Materials and Methods

### 2.1. Samples

**Figure 1:**
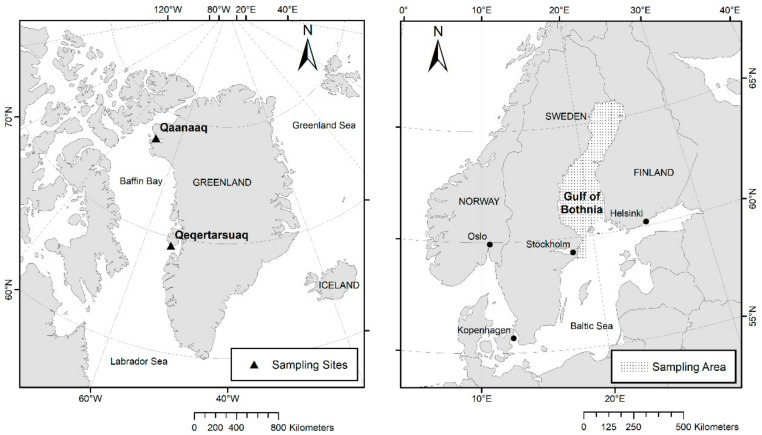
Examined Area. The collection of the samples originates from the Gulf of Bothnia in the Baltic Sea and in West Greenland. The samples were predominantly collected in the locations marked with a circle in the Gulf of Bothnia and with a triangle at the west coast of Greenland.

The samples used in this study originated from ringed seals that either died through stranding or were legally hunted by local hunters in Sweden, Finland, and Greenland. The animals were shot according to the existing hunting law in the respective countries. A total of 52 juvenile ringed seal ovaries from the Gulf of Bothnia, a region in the Baltic Sea and the west coast of Greenland were collected and examined (Figure 1).

**Figure 2:**
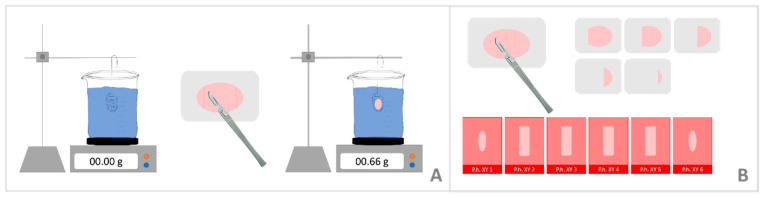
Setup for the volume determination of each ovary (**A**). After complete immersion of the ovary in water, the displaced volume of water corresponds to the volume of the ovary and can be measured as the weight change. The histological preparation for each ovary is shown in (**B**). Cross sections of the ovary were made.

The 46 ovaries from the Gulf of Bothnia, including 13 ovaries from Swedish individuals and 33 Finnish individuals, were provided from the Swedish Museum of Natural History in Stockholm (SMNH) and from the Natural Resources Institute in Finland (LUKE), the six ovaries from Greenland were provided from the Aarhus University in Denmark. All ovaries were collected between 2018 and 2019. The age of 43 individuals was determined based on the pupping season in the different areas (Baltic Sea: between February and March [27], Greenland: between March and early April [15]), for nine individuals the date of death was missing. The age of 43 individuals was approximately between 1.5 and 2.5 months. The ovaries were stored in a 4% phosphate-buffered formalin solution, whereas beforehand most ovaries were stored frozen at −20 °C for up to nine months. For microscopic examination, one ovary per animal, mostly the left (in one case the right ovary), was chosen for further investigations. The total volume of each ovary was determined by fluid displacement method (Principle of Archimedes) [28] (Figure 2A). After complete immersion of the ovary in water, the displaced volume of water corresponds to the volume of the ovary and can be measured by the weight change. Measurements were repeated three times for each ovary and the average was calculated.

### 2.2. Histology Preparation

Each ovary was cut exhaustively into 2 mm thick slices, starting at a random position between 0 and 2 mm as a starting point for the first cut. After examination of the slices for the presence of tertiary follicles, if none were found (Figure 2B) every slice was embedded in paraffin using standard methods (Leica Paraplast Standard) for a detailed investigation. From each of the embedded slices, one 5 µm thick section was cut and stained with haematoxylin-eosin (HE) (N = 170). Due to the random starting position during the cutting of the ovary, the sampling procedure fulfills the criteria of systematic uniform random sampling, thus giving each part of the organ an equal chance of being included in the analysis.

### 2.3. Stereology—Total Primordial Follicle Number

**Figure 3:**
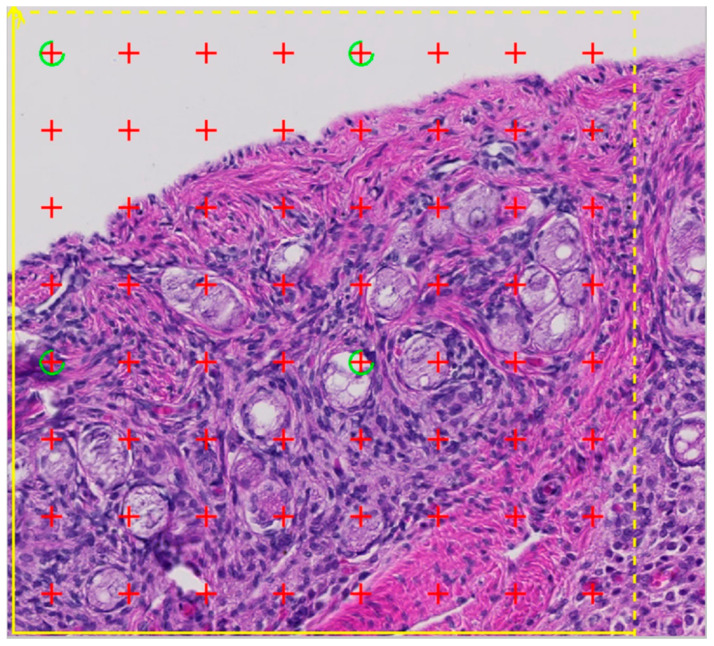
A part of the stained ovary slice with the test grid of 64 points. Points hitting the primordial follicles as well as the four green-colored points hitting the reference space (ovary) were counted and afterwards multiplied with 16 (coarse grid).

The aim of stereology is to obtain quantitative information about the three-dimensional architecture of an organ by analyzing plane, two-dimensional tissue sections [29]. In this study, the total volume of primordial follicles per ovary and the mean volume of the follicles were estimated by stereology. The total number of primordial follicles was calculated from these two parameters. From each ovarian slice one section was digitized using a slide scanner (Axio Scan.Z1, ZEISS, Oberkochen, Germany). Images were subsampled using the newCast software (Visiopharm, Hørsholm, Denmark) and analyzed with the newCast software (Visiopharm, Hørsholm, Denmark) and the STEPanizer online tool [30]. The total volume of primordial follicles per ovary was determined by point counting. The sampling fraction was set as 20% and an objective lens magnification of 20× was used. The test grid consisted of 64 evenly arranged points (Figure 3), four of which were marked green and enclosed exactly 25% of the total viewed area in a rectangular shape. The fine grid was used for the estimation of the primordial follicles, i.e., points of the fine grid hitting the primordial follicles (*P(follicle)*) were counted. The coarse grid (green-colored points) were used to assess the volume of the reference space, i.e., green-colored points hitting the ovarian tissue (*P(ovary)*) were counted. Afterwards the points hitting the ovary (*P(ovary)*) were multiplied by 16, as each of the green-marked points represented one quarter of the total number of points (64).

The volume density of the primordial follicles (*Vv(follicle/ovary)*) was calculated by dividing *P(follicle)* by *P(ovary)* (Formula (1)).

(1) $$V_{v}\left(follicle/ovary\right)=\frac{P\left(follicle\right)}{P\left(ovary\right)}$$

The total volume of follicles ($V\left(follicle,\;ovary\right))$, was computed by multiplication of the volume density by the total volume of the ovary (Formula (2)) (Table S1).

(2) $$V\left(follicle,\;ovary\right)=V_{v}\left(follicle/ovary\right)\times V\left(ovary\right)$$

In the next step, the number-weighted mean volume of the follicles (${\bar{V}}_{N}\left(follicle\right)$) was estimated using the rotator [12,31,32] (Table S1). Here, a single section dissector was used to sample follicles for volume measurements instead of a physical or optical dissector [33]. During testing of the different methods, it became clear that the status of the tissue preservation made a formally more correct approach hardly possible in this rare material. The planar rotator was used every time a centrally sectioned primordial follicle crossed the green lines or was completely within the counting frame and did not touch the red exclusion lines or their extensions.

**Figure 4:**
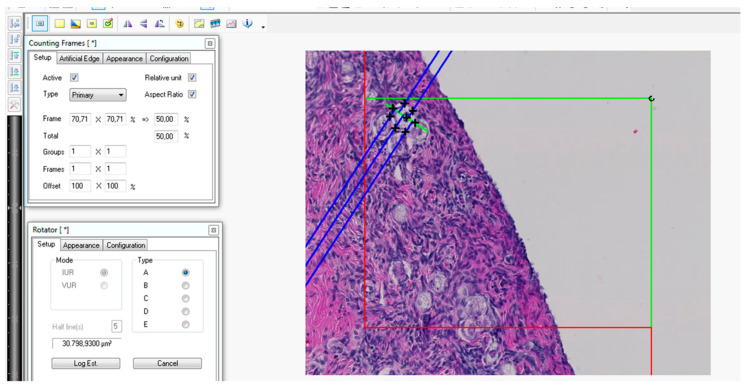
Volume determination of different primordial follicles. All central sectioned primordial follicles, which cross the green line or are inside the marked area are taken into account for volume measurements.

Finally, the total number of primordial follicles was calculated by dividing the total volume of follicles ($V\left(follicle,\;ovary\right)$) by the number-weighted mean volume of primordial follicles (${\bar{V}}_{N}\left(follicle\right),\;$ Figure 4). *N (follicle, ovary)* is the total estimated number of follicles per ovary (Table S1).

(3) $$N\left(follicle,\;ovary\right)=\frac{V\left(follicle,\;ovary\right)}{{\bar{V}}_{N}\left(follicle\right)}$$

### 2.4. Statistical Analyses

The statistical analyses were performed with R (version 3.4.3) [34]. Due to a small sample size and a large variability in the calculated total number of primordial follicles (*N(follicle, ovary)*), the effects of different regions (Baltic Sea, Greenland) on the number of primordial follicles was explored descriptively. In this context, boxplots were generated for comparison. Values are reported as median and the interquartile range (IQR).

Furthermore, a linear regression analysis (LM) was conducted, where the relationship of the number-weighted mean volume of the primordial follicles, the volume density of follicles (*Vv(follicle/ovary)*) and the total follicle volume (*V(follicle, ovary)*) were tested against the examined areas. Additionally, the relationship of the ovary volume (*V(ovary)*) and the number-weighted mean volume (${\bar{V}}_{N}\left(follicle\right)$) were tested against each other in a separate LM.

## 3. Results

### 3.1. Number of Primordial Follicles and Geographical Differences

**Figure 5:**
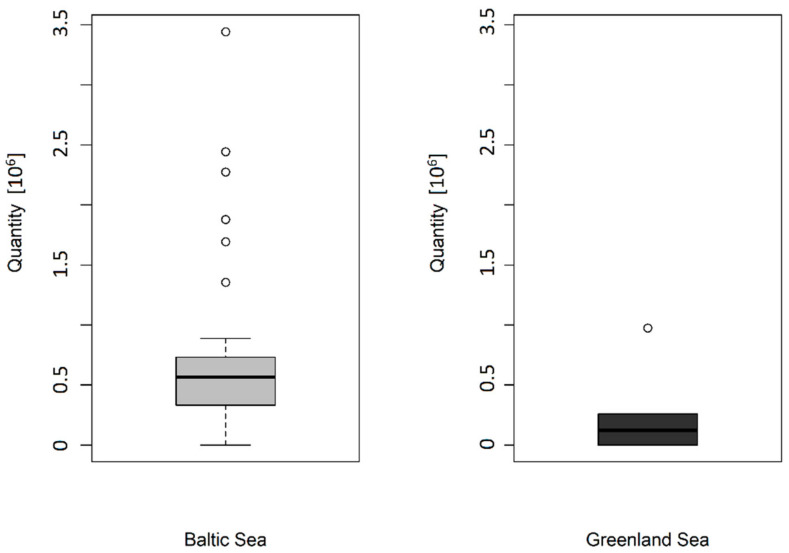
Total primordial follicle number of Baltic and Greenland ringed seals. It is shown that the quantity of primordial follicles in seals from the Baltic Sea varies more.

The total estimated number of primordial follicles was analyzed in 46 ovaries from juvenile ringed seals from the Baltic Sea, (divided into 13 Swedish individuals and 33 Finish individuals) and six ovaries from Greenland individuals. Figure 5 shows the median primordial follicle number in the Baltic and in the Greenland individuals (median: Baltic Sea = 565,657; IQR: 399,602; Greenland Sea = 122,475; IQR: 202,096; no statistical test was performed due to low sample size). Unfortunately, it is not possible to make a comparison between the two subpopulations, because the data basis of the Greenlandic individuals is not sufficient.

### 3.2. Volume Differences

No significant differences in the number-weighted mean volume of primordial follicles could be found between the two areas. A negative but not significant trend could be found between the volume of ovaries of the Greenland individuals (mean 0.62 cm^3^) and the ovary volume of Baltic individuals (mean 0.95 cm^3^, *p* = 0.096).

The medians of the volumes differ between the two ringed seal populations. The ovary volume (V(ovary)), number-weighted mean volume (${\bar{V}}_{N}\left(follicle\right)$) (mean_Greenland_ 1.66 × 10^−8^ cm^3^; mean_Baltic Sea_ 9.48 × 10^−9^ cm^3^), volume density of follicles (*Vv(follicle/ovary)*) (mean_Greenland_ 0.0048 cm^3^; mean_Baltic Sea_ 0.0057 cm^3^), and total follicle volume (*V(follicle, ovary)*) (mean_Greenland_ 0.0035 cm^3^; mean_Baltic Sea_ 0.0051 cm^3^) were lower in Greenland individuals than Baltic individuals.

**Figure 6:**
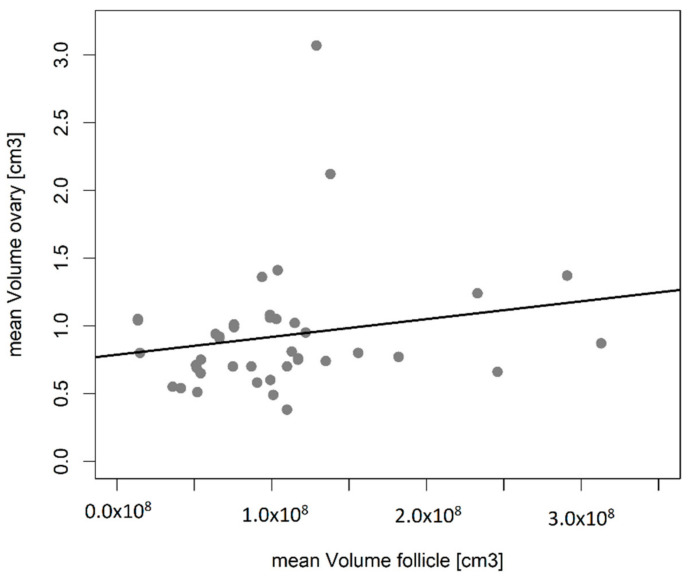
Correlation between number-weighted mean volume of follicles and ovary volume in juvenile ringed seals. The number-weighted mean volume shows a significant positive relationship with the ovary volume (LM, *p* = 0.04).

A positive correlation between the number-weighted mean volume of follicles and volume of ovaries were found (LM, *p* = 0.04) (Figure 6).

## 4. Discussion

### 4.1. Identification of Primordial Follicle Number

The total primordial follicle number in the ovaries of ringed seals from Greenland and the Gulf of Bothnia varies between the areas. It is not possible to make a comparison between the two subpopulations, because the data basis of the Greenlandic individuals is too low. The same goes for primordial follicle numbers in this population. In the Gulf of Bothnia, the data show that the primordial follicle numbers are between 138,078 and 3,441,696. The outliners of the total primordial follicle number, which can be seen in Figure 5, underline the high variability in primordial follicle numbers in individuals of the Gulf of Bothnia. Furthermore, the number of ringed seal follicles in total was markedly higher (mean of all examined ovaries: 659,273 in one ovary) than the primordial follicle numbers of other species after birth, like bovines (*Bos taurus*), dogs (*Canis lupus familiaris*), pigs (*Sus scrofa domesticus*), or mice (*Mus musculus*) [35,36,37,38,39] (Table 1).

It is known that the number of primordial follicles in larger species (e.g., dogs or humans (*Homo sapiens*) is higher, because of a greater adult life span [40] and the natural gradual decline with age, which begins with a reduced fertility and ends with a natural sterility [12]. In addition, the surplus provision of follicles represents a wide range of safety measures, which can become important for preservation of genetic material, when the individuals have an abortion or no successful first fertilization [41]. Ringed seals can have such problems during the whole reproductive cycle, because the cycle is sensitive and influenced by different factors, such as prey availability, body condition, anthropogenic substances, or climate conditions [18,26]. Ringed seals ovulate only once per year, in the breeding season during spring. In case of a failure in fertilization, it is possible that a second ovulation takes place in the same season [7]. Furthermore, young individuals, which ovulate for the first time, can also have double ovulations [7]. Female ringed seals reach sexual maturity between four and six years [42], which is later than, for example, in bovines, dogs, and pigs (see Table 1). The development of primordial follicles starts directly after birth. Ringed seals probably lose a lot of primordial follicles until they reach sexual maturity, as well as humans, because the exact year of sexual maturity can be influenced by different environmental conditions. A comparably late sexual maturity implies a greater loss of primordial follicles before puberty, which can also explain the higher primordial follicle numbers in ringed seals, than in other mammals. This can be compared with humans, who also have a high pool of primordial follicles and lose many of them during their lifetime (before puberty and with increasing age). The obvious differences between the primordial follicle numbers of larger species, given in Table 1, and the results of the examined ringed seals in this study, can be explained and/or biased by the usage of different methods, but also by natural, ecological, and behavioral differences and the fact that ringed seals can have pups even when they are over 20 years old [9]. It is conceivable, that the primordial follicle number of the ringed seals in this study is very high due to the inclusion of primordial follicles that are not totally developed. Thus, these follicles are potentially lost until sexual maturity [40]. Another reason could be the use of paraffin sections. The tissue shrinks in paraffin by about 40 to 60% depending on the water content. Thus, the number is probably overestimated here. Furthermore, the species in Table 1 are different as some cycle all year round while others are seasonal, some have one offspring and others have more (pigs, dogs, and rats).

Ringed seals can be divided into different subpopulations [15,16,17,18]. The Gulf of Bothnia individuals examined in this study represent the Baltic ringed seal subpopulation, whereas the Greenland individuals represent the Arctic ringed seal subpopulation. Subpopulations commonly differ in the timing of breeding season or adaptions to their direct environment [15,27]. The Greenlandic ringed seals struggle with ice loss, which lead to a higher stress level for these individuals [43]. Furthermore, increasing levels of different pollutants (e.g., PCB, PFAS) have an effect on the overall health of the Arctic ringed seals [44]. Overall, there are certain aspects, which could affect the primordial follicle, although no conclusion can be drawn about the Greenland population. Therefore, it is of great relevance to gather more data from this population to define the exact primordial follicle number and compare that to the Gulf of Bothnia subpopulation.

An explanation for the differences between both subspecies is the different number of examined individuals that might bias the results and their relation. More individuals of the Gulf of Bothnia, a region of the Baltic Sea (N = 46) were investigated in relation to Greenland individuals (N = 6). The lower primordial follicle numbers of Arctic ringed seals can be explained by the low number of examined individuals. The low number of examined individuals does not represent a good baseline of primordial follicles for the whole Arctic ringed seals. Further examinations are needed with a larger number of Arctic ringed seals to verify the differences in primordial follicle numbers and to compare both subspecies.

### 4.2. Volume Differences

The mean ovary volume differed between ringed seal individuals from the Gulf of Bothnia and west Greenland. Again, it is not possible to make a comparison between the two subpopulations, because the data basis of the Greenlandic individuals is too low. The mean ovary volume of ringed seals was between 0.33 and 0.66 cm^3^. Older ringed seals have bigger ovaries than juveniles, because they have follicular waves, *corpa lutea,* and/or *corpora albicantia*. The investigated ovary volume might change with body size, weight, and season. There are no reference values to compare these results with other seal species and an assessment is therefore not possible. The same applies to the number-weighted mean volume of follicles, which differed slightly with respect to the mean values of the individuals from both areas. The volume density of the follicles in the individuals of the Gulf of Bothnia seems to be higher than in individuals of Greenland.

Overall, the study serves as a baseline for following studies and states some first detailed evaluation of the ovaries from remote species. Based on this, future investigations that utilize the same methods have the potential to reveal changes and thus, build a bridge to the understanding of wild life species in a changing and human influenced environment.

## 5. Conclusions

In contrast to terrestrial and laboratory mammals, the acquisition of fresh ovarian material is difficult. Hence, the tissue preservation and the application of morphometric/stereological methods is challenging. Here, we have provided a suitable methodological compromise between feasibility and stereological correctness to obtain volumetric and numeric data on ringed seal ovaries. In future studies, it is important to have a quick and direct transfer of the ovaries to formalin fixative as it would allow a formally more correct stereological approach (dissector, fractionator).

Nevertheless, our study provides the first reference data of primordial follicles in ringed seals of different origin. The great variability of the data may have a biological or methodological origin, or could possibly reflect an influence of environmental factors. Further investigations need to be conducted with more individuals and further developed methodology. Overall, there appear to be differences in follicle number and volume with respect to different geographic origin. This data needs to be taken into account in future studies investigating habitat changes when seals with different geographic background are grouped together.

## Figures and Tables

**Table 1:** Primordial follicle number of different species in both ovaries.

Species	Primordial Follicle Number—Birth	Primordial Follicle Number—Puberty	Age of Sexual Maturity
Bovines (*Bos taurus*) ^a^	~100,000	~20,000	8–10 months
Human (*Homo sapiens*) ^b^	~1,000,000–2,000,000	~300,000–400,000	8–13.5 years
Dog (*Canis lupus familiaris*) ^c^	~700,000	~350,000	6–12 months
Pig (*Sus scrofa domesticus*) ^d^	~500,000	~200,000	~6 months
Mouse (*Mus musculus*) ^e^	10,000–15,000	u.	~28 days

^a^ [35]; ^b^ [36]; ^c^ [37]; ^d^ [38]; ^e^ [39]; u. = unknown.

## Data Availability

The data presented in this study is available on request from the corresponding author. The data is not publicly available because it is part of the data collected in the context of a project to which only the institutes involved in the project have direct access.

## Supplementary Materials

The following are available online at https://www.mdpi.com/article/10.3390/ani12050669/s1, Table S1: All points hitting primordial follicles *P(follicle)* and all points hitting the ovary P *P(ovary)* were counted for the ovaries of ringed seals from three different countries. As a result of Formula 1, the volume density *Vv(follicle/ovary)* was calculated with *P(follicle)* and *P(ovary)*. *Vv(follicle/ovary)* and *V(ovary)*, the volume of the ovary, were used to calculate the total volume of follicles in the ovary *V(follicle, ovary)* based on Formula 2. Using this value and number-weighted mean volume of follicles *VN(follicle)*, the total number of follicles in the ovary was determined.
