# Supplementary material for: Number of Primordial Follicles in Juvenile Ringed Seals (Pusa hispida) from the Gulf of Bothnia and West Greenland
Source: Animals (Basel). 2022 Mar 7;12(5):669. doi: 10.3390/ani12050669 (PMC8909318; doi:10.3390/ani12050669)
Supplement: Supplementary file 1 [file animals-12-00669-s001.zip › animals-1588640-supplementary.pdf]

Supplementary Material

Table S1. All points hitting primordial follicles P(follicle) and all points hitting the ovary P P(ovary) were counted for the ovaries of ringed seals from three different countries. As a result of Formula 1, the volume density V_v(follicle/ovary) was calculated with P(follicle) and P(ovary). V_v(follicle/ovary) and V(ovary), the volume of the ovary, were used to calculate the total volume of follicles in the ovary V(follicle, ovary) based on Formula 2. Using this value and number-weighted mean volume of follicles V_N(follicle), the total number of follicles in the ovary was determined.

**Individuals**	**Age**	**Country**	**P (follicle)**	**P (ovary)**	**Vv (follicle/ovary) [%]**	**V (ovary)** [cm^3]	**V(follicle, ovary)** [cm^3]	**V_N (follicle)** [cm^3]	**N(follicle, ovary)**
P.h. 1		Sweden	37	6544	5.654	0.99	$56.0*10^{-4}$	$7.58*10^{-9}$	738,456
P.h. 2		Sweden	20	10576	1.891	1.62	$30.6*10^{-4}$	NA	NA
P.h. 3		Sweden	12	5376	2.232	0.65	$14.5*10^{-4}$	$5.41*10^{-9}$	268,188
P.h. 4		Sweden	9	9360	0.962	1.09	$10.5*10^{-4}$	NA	NA
P.h. 5		Sweden	53	11776	4.501	1.04	$46.8*10^{-4}$	$1.36*10^{-9}$	3,441,696
P.h. 6		Sweden	60	11728	5.116	1.08	$55.3*10^{-4}$	$9.90*10^{-9}$	558,105
P.h. 7		Sweden	20	8144	2.456	1.04	$25.5*10^{-4}$	$1.36*10^{-9}$	1,877,961
P.h. 8		Sweden	28	6304	4.442	0.92	$40.9*10^{-4}$	$6.63*10^{-9}$	616,334
P.h. 9		Sweden	16	15440	1.036	1.,01	$10.5*10^{-4}$	$7.58*10^{-9}$	138,078

P.h. 10	2.5	Finland	143	13312	10.742	1.24	$133.2*10^{-4}$	$23.31*10^{-9}$	571,442
P.h. 11	2.5	Sweden	226	14176	15.942	1.37	$218.4*10^{-4}$	$29.10*10^{-9}$	750,555
P.h. 12	2.5	Sweden	184	8752	21.024	0.77	$161.9*10^{-4}$	$18.20*10^{-9}$	889,467
P.h. 13	2.5	Sweden	70	8512	8.224	0.66	$54.3*10^{-4}$	$24.63*10^{-9}$	220,367
P.h. 14	1.5	Finland	24	16768	1.431	1.74	$24.9*10^{-4}$	NA	NA
P.h. 15	1.5	Finland	28	7264	3.855	1.01	$38.9*10^{-4}$	$7.58*10^{-9}$	513,611
P.h. 16	1.5	Finland	30	6896	4.350	0.94	$40.9*10^{-4}$	$6.38*10^{-9}$	640,960
P.h. 17	1.5	Finland	83	27824	2.983	3.07	$91.6*10^{-4}$	$12.90*10^{-9}$	709,916
P.h. 18	1.5	Finland	11	5328	2.065	0.55	$11.4*10^{-4}$	$3.60*10^{-9}$	315,420
P.h. 19	1.5	Finland	39	17072	2.284	2.12	$48.4*10^{-4}$	$13.75*10^{-9}$	352,219
P.h. 20	2	Finland	44	6480	6.790	0.80	$54.3*10^{-4}$	$15.63*10^{-9}$	347,543
P.h. 21	2.5	Finland	31	6768	4.580	0.80	$36.6*10^{-4}$	$1.50*10^{-9}$	2,442,868
P.h. 22	2	Finland	25	5648	4.426	0.65	$28.8*10^{-4}$	$5.40*10^{-9}$	531,816
P.h. 23	2	Finland	58	10976	5.284	1.36	$71.9*10^{-4}$	$9.38*10^{-9}$	766,161
P.h. 24	2	Finland	8	4336	1.845	0.51	$9.4*10^{-4}$	$5.20*10^{-9}$	180,954

P.h. 25	2	Finland	58	4144	13.996	0.49	$68.6*10^{-4}$	$10.12*10^{-9}$	677,679
P.h. 26	1.5	Finland	103	8144	12.647	1.06	$134.1*10^{-4}$	$9.90*10^{-9}$	1,354,160
P.h. 27	2	Finland	22	7312	3.009	0.91	$27.4*10^{-4}$	$6.63*10^{-9}$	412,966
P.h. 28	1.5	Finland	43	6832	6.294	0.76	$47.8*10^{-4}$	$11.67*10^{-9}$	409,886
P.h. 29	2	Finland	28	3824	7.322	0.38	$27.8*10^{-4}$	$11.00*10^{-9}$	252,948
P.h. 30	2	Finland	61	11904	5.124	1.41	$72.3*10^{-4}$	$10.44*10^{-9}$	692,079
P.h. 31	2.5	Finland	32	5536	5.780	0.58	$33.5*10^{-4}$	$9.06*10^{-9}$	370,044
P.h. 32	2.5	Finland	30	6672	4.496	0.71	$31.9*10^{-4}$	$5.11*10^{-9}$	624,745
P.h. 33	2.5	Finland	13	8064	1.612	1.05	$16.9*10^{-4}$	$10.27*10^{-9}$	164,821
P.h. 34	2	Finland	61	8080	7.550	0.81	$61.2*10^{-4}$	$11.34*10^{-9}$	539,250
P.h. 35	2.5	Finland	28	9504	2.946	1.05	$30.9*10^{-4}$	$1.36*10^{-9}$	2,274,584
P.h. 36	2.5	Finland	41	4384	9.352	0.60	$56.1*10^{-4}$	$9.92*10^{-9}$	565,657
P.h. 37	2	Finland	14	5872	2.384	0.70	$16.7*10^{-4}$	$10.96*10^{-9}$	152,275
P.h. 38	2	Finland	1	2400	0.417	0.36	$1.5*10^{-4}$	0	0

P.h. 39	2.5	Finland	0	4768	0	0.56	0	0	0
P.h. 40	2.5	Finland	27	5568	4.849	0.69	$33.5*10^{-4}$	$5.16*10^{-9}$	648,431
P.h. 41	2	Finland	46	5120	8.984	0.70	$62.9*10^{-4}$	$8.69*10^{-9}$	723,713
P.h. 42	2	Finland	12	5168	2.322	0.54	$12.5*10^{-4}$	$4.13*10^{-9}$	303,600
P.h. 43	2	Finland	68	5552	12.248	0.75	$91.9*10^{-4}$	$5.43*10^{-9}$	1,691,690
P.h. 44	2.5	Finland	89	8208	10.843	0.95	$103.0*10^{-4}$	$12.19*10^{-9}$	845,031
P.h. 45	1.5	Finland	35	4528	7.730	0.70	$54.1*10^{-4}$	$7.50*10^{-9}$	721,437
P.h. 46	1.5	Finland	27	4528	5.963	1.02	$60.8*10^{-4}$	$11.50*10^{-9}$	528,883
P.h. 47	1.5	Greenland	144	9504	15.152	0.75	$113.6*10^{-4}$	$11.67*10^{-9}$	973,748
P.h. 48	1.5	Greenland	48	9728	4.934	0.87	$42.9*10^{-4}$	$31.30*10^{-9}$	137,149
P.h. 49	1.5	Greenland	56	11856	4.723	0.74	$35.0*10^{-4}$	$13.46*10^{-9}$	259,679
P.h. 50	1.5	Greenland	15	8416	1.782	0.6	$10.7*10^{-4}$	$9.92*10^{-9}$	107,802
P.h. 51	1.5	Greenland	7	3920	1.786	0.33	$5.9*10^{-4}$	0	0
P.h. 52	1.5	Greenland	3	5088	0.590	0.4	$2.4*10^{-4}$	$10.12*10^{-9}$	23,305

* NA = not available, too small P(follicle) values
